# Risk of aortic aneurysm and dissection in patients with autosomal-dominant polycystic kidney disease: a nationwide population-based cohort study

**DOI:** 10.18632/oncotarget.16338

**Published:** 2017-03-17

**Authors:** Pei-Hsun Sung, Yao-Hsu Yang, Hsin-Ju Chiang, John Y. Chiang, Chi-Jen Chen, Chien-Ting Liu, Cheuk-Man Yu, Hon-Kan Yip

**Affiliations:** ^1^ Department of Internal Medicine, Division of Cardiology, Kaohsiung Chang Gung Memorial Hospital and Chang Gung University, College of Medicine, Kaohsiung, Taiwan; ^2^ Department of Traditional Chinese Medicine, Chang Gung Memorial Hospital, Chiayi, Taiwan; ^3^ Institute of Occupational Medicine and Industrial Hygiene, College of Public Health, National Taiwan University, Taipei, Taiwan; ^4^ Department of Obstetrics and Gynecology, Kaohsiung Chang Gung Memorial Hospital and Chang Gung University, College of Medicine, Kaohsiung, Taiwan; ^5^ Department of Computer Science and Engineering, National Sun Yat-Sen University, Kaohsiung, Taiwan; ^6^ Department of Healthcare Administration and Medical Informatics, Kaohsiung Medical University, Kaohsiung, Taiwan; ^7^ Center of Excellence for Chang Gung Research Datalink, Chang Gung Memorial Hospital, Chiayi, Taiwan; ^8^ Department of Hematology-Oncology, Kaohsiung Chang Gung Memorial Hospital and Chang Gung University, College of Medicine, Kaohsiung, Taiwan; ^9^ Director of Heart Centre, Hong Kong Baptist Hospital and Honorary Clinical Professor, The Chinese University of Hong Kong, Hong Kong, China; ^10^ Institute for Translational Research in Biomedicine, Center for Shockwave Medicine and Tissue Engineering, Kaohsiung Chang Gung Memorial Hospital and Chang Gung University, College of Medicine, Kaohsiung, Taiwan; ^11^ Department of Medical Research, China Medical University Hospital, China Medical University, Taichung, Taiwan; ^12^ Department of Nursing, Asia University, Taichung, Taiwan

**Keywords:** autosomal-dominant polycystic kidney disease, aortic aneurysm, aortic dissection, population-based cohort study

## Abstract

Although cardiovascular complications are the most common cause of death in patients with autosomal-dominant polycystic kidney disease (ADPKD), the incidence and risk of aortic aneurysm and dissection (AAD) in ADPKD remains unclear due to limited data and insufficient cases. We utilized the data from Taiwan National Health Insurance Research Database (NHIRD) to do a population-based cohort study (1997-2008). After excluding those patients with age <18 years old and initially concomitant diagnoses of end-stage renal disease and AAD, a total of 2076 ADPKD patients were selected from 1,000,000 of general population. Additionally, the non-ADPKD group was set up as comparison group in 1:10 ratio after matching with age, gender, income and urbanization (*n*=20760). The result showed that ADPKD group had higher frequency of comorbidities than non-ADPKD group. The frequency of AAD in ADPKD was significantly higher than in general population (0.92% v.s. 0.11%, *p*<0.0001). Of them, 58% of AAD were acute aortic dissection. In addition, Kaplan-Meier analysis demonstrated that cumulative incidence of AAD was remarkably higher in the ADPKD than non-ADPKD group (*p*<0.001). The mean time period from ADPKD diagnosis to AAD occurrence was 4.02±3.16 years. After adjusting for age, gender and comorbidities, the ADPKD patients had up to 5.49-fold greater risk for AAD occurrence as compared to non-ADPKD counterparts (95% CI 2.86-10.52, *p*<0.0001). Particularly, those patients with co-existing ADPKD and hypertension had very high risk for future development of AAD. In conclusion, the risk of AAD significantly increases in patients with ADPKD as compared with those of general population.

## INTRODUCTION

Autosomal-dominant polycystic kidney disease (ADPKD) is the most common inherited kidney disease, with the incidence ranging from 1/400 to 1/1000 in United States [1, 2]. The ADPKD is genetically heterogeneous, and mutation of PKD 1 and PKD 2 genes contributes to its development. Without appropriate treatment, an estimation of 2-5% patients with ADPKD would eventually progress to end-stage renal failure [3, 4]. Studies have previously revealed that vascular manifestations of ADPKD are pathological dolichoectasias (i.e., elongations and distentions of the arteries caused by weakening of the vessel walls) and dissections [5–7]. Additionally, polycystin 1 and polycystin 2, two essential protein products of PKD1 and PKD2 respectively, are expressed in vascular smooth muscle and endothelium. Thus, interactions of these proteins with a hereditary pathological pathway might be the underlying mechanism involved in the early development of vascular remodeling and aneurysms in ADPKD [5–7]. This could be explained for why many extrarenal manifestations, especially in those of cardiovascular (CV) and cerebrovascular abnormalities, are found to co-exist with ADPKD. Further detailed analysis has demonstrated that the left ventricular hypertrophy, cardiac valvular defects, and intra- or extra-cranial aneurysms are majority of these complications [6]. Importantly, studies have previously further identified that CV complications are the most common causes of morbidity and mortality in patients with ADPKD [8].

Interestingly, although the frequency of intracranial aneurysm in patients with ADPKD has been reported up to 4-11% [9, 10], little is known regarding the frequency of extracranial aneurysm in this inherited kidney disease [5, 10–12], especially those of aortic aneurysm, aortic dissection, or coexistence of both conditions. They should deserve more concern since urgent management or emergent surgery for aneurysmal dissection/aortic dissection is mandatory for life-saving if early recognition and diagnosis is achieved. Furthermore, although the prevalence of aortic aneurysm/dissection (AAD) in setting of ADPKD has previously been investigated by limited studies, the results are discrepant [13–16], likely due to the small sample size. Moreover, there is also lack of relevant studies on the incidence of AAD in ADPKD. This raises the need of a further study with a larger sample size to delineate the real-world incidence and associated risk of AAD in patients with ADPKD.

Taiwan National Health Insurance Research Database (NHIRD) is a nationwide population-based database that covers 99% of all Taiwan residents and provides comprehensive medical information. The database has widely been used for epidemiological research [17, 18] and information on diagnoses and hospitalizations has also been proven to be high quality and scientific, especially when applied in catastrophic illnesses. Therefore, by using the NHIRD with a 12-year period, we conducted a nationwide cohort study to investigate whether ADPKD increases the risk of AAD.

## RESULTS

### Characteristics of the study participants (Table 1)

The baseline characteristics of study patients and matched control subjects are shown in Table 1. A total of 2,076 patients with ADPKD and 20,760 matched patients without ADPKD were eligible during 12-year dataset period. The results demonstrated that 51.64% patients were female and median age was 47 years old (interquartile range 38-56) in both ADPKD and non-ADPKD groups. Most of the patients were middle-aged (40-65 years old, 61.5%) and had urbanization level 1-2 (80.11%) in both groups. In addition, majority of their economic status was well-fixed (69.12%) and identical in both groups. Of note, the frequency of atherosclerotic risk factors (i.e., hypertension, dyslipidemia, gout, and chronic kidney disease) and comorbidities (i.e., atrial fibrillation, ischemic heart disease, heart failure, stroke, peripheral vascular disease, and urogenital malignancy) was remarkably higher in the ADPKD group than that of the non-ADPKD group (all *p*-values < 0.002), except for diabetes mellitus (14.93% v.s. 15.02%, *p* = 0.9114).

**Table 1:** Demographic characteristics and frequency of medical diseases in patients with ADPKD and without ADPKD

	ADPKD (*N* = 2,076)		Non-ADPKD* (*N* = 20,760)		
	No.	%	No.	%	*p*-value^†^
Gender					1.00
Female	1072	51.64	10720	51.64	
Male	1004	48.36	10040	48.36	
Age					1.00
18-39	575	27.7	5750	27.7	
40-65	1276	61.46	12760	61.46	
>65	225	10.84	2250	10.84	
Median age (IQR)	47 (38-56)		47 (38-56)		
Urbanization level					1.00
1 (highest)	748	36.03	7480	36.03	
2	915	44.08	9150	44.08	
3	295	14.21	2950	14.21	
4 (lowest)	118	5.68	1180	5.68	
Income (NTD per month)					1.00
0	344	16.57	3440	16.57	
1-15,840	297	14.31	2970	14.31	
15,841-25,000	869	41.86	8690	41.86	
>25,000	566	27.26	5660	27.26	
Medical diseases					
Hypertension	1684	81.12	6572	31.66	<.0001
Diabetes mellitus	310	14.93	3119	15.02	0.9114
Dyslipidemia	718	34.59	4672	22.5	<.0001
Gout	590	28.42	2557	12.32	<.0001
Atrial fibrillation	48	2.31	295	1.42	0.0015
Ischemic heart disease	541	26.06	3265	15.73	<.0001
Heart failure	220	10.6	822	3.96	<.0001
Cerebrovascular accident	319	15.37	1522	7.33	<.0001
Hemorrhagic stroke	121	5.83	249	1.2	<.0001
Peripheral vascular disease	136	6.55	671	3.23	<.0001
Chronic kidney disease	1025	49.37	470	2.26	<.0001
Malignancy of kidney or bladder	66	3.18	88	0.42	<.0001
AAD					<.0001^‡^
No	2057	99.08	20738	99.89	
Yes	19	0.92	22	0.11	

*Control group (non-ADPKD) was matched by age, sex, monthly income and urbanization level.

^†^Chi-square test for categorical variables, ^‡^Fisher Exact test.

Abbreviations: AAD, aortic aneurysm/dissection; ADPKD, autosomal-dominant polycystic kidney disease; IQR, interquartile range; NTD, New Taiwan dollars.

At the end of follow-up period, a total of 19 and 22 cases of AAD developed in 2,076 ADPKD and 20,760 non-ADPKD patients, respectively. Therefore, the frequency of AAD was significantly higher in the ADPKD than non-ADPKD group (0.92% v.s. 0.11%, *p* < 0.0001) (Table 1).

Of these 19 cases of AAD in ADPKD, 8 patients had aortic aneurysm and the other 11 patients (58%) had acute aortic dissection. We also noted higher rate of thoracic aneurysm than abdominal aneurysm. However, lesion location could not be further exactly described in one patient with aortic aneurysm and 8 patients with aortic dissection. This was because a part of the ICD-9-CM codes entered were undefined. On the other hand, a total of 22 patients developed AAD in the non-ADPKD group. Of them, 14 patients had aortic aneurysm and the other 8 patients had aortic dissection. Therefore, different from majority (58%) of ADPKD patients having complicated aortic dissection, the ratio of aortic dissection to AAD in non-ADPKD group was relatively low (36%) (Supplemental Table 1).

### Comparison of incidence and associated risk of AAD between patients with ADPKD and without ADPKD (Table 2)

The incidence rate of AAD in patients with and without ADPKD was 142.7 and 15.7 per 100,000 person-years, respectively. Thus, incidence rate ratio (IRR) of AAD in ADPKD to non-ADPKD was 9.11 (95% CI 4.93 to 16.83, *p* < 0.0001). After adjusting for age, gender and medical diseases with multivariate Cox regression analysis, patients with ADPKD had 5.49-fold risk for future development of AAD as compared with those non-ADPKD patients (95% CI 2.86 to 10.52, *p* < 0.0001). Besides, stratified analysis which was utilized to clarify the impact of each comorbidity on AAD demonstrated that adjusted hazard ratio (HR) for the incidence of AAD in ADPKD to non-ADPKD group was significantly higher in the subgroup of both gender, age ≥40 years old, and those patients with hypertension, diabetes, dyslipidemia, ischemic heart disease and cerebrovascular accident (all *p*-values < 0.04). These findings suggested that as to patients aged ≥40 years old, ADPKD could be identified as an independent risk factor for AAD regardless of their gender and comorbidities.

**Table 2:** Comparison of incidence and hazard ratio of AAD between patients with and without ADPKD, stratified by gender, age and medical diseases

	ADPKD group (*N* = 2076)			Non-ADPKD group (*N* = 20760)				
Subgroup	AAD	PY	Rate	AAD	PY	Rate	IRR (95% CI)	Adjusted HR (95% CI)
Total	19	13312	142.7	22	140417	15.7	9.11 (4.93 - 16.83)^‡^	5.49 (2.86 - 10.52)^‡^
Gender								
Female	5	7041	71	7	73581	9.5	7.46 (2.37 - 23.52)^‡^	5.22 (1.53 - 17.86)^†^
Male	14	6271	223.2	15	66836	22.4	9.95 (4.8 - 20.61)^‡^	5.41 (2.51 - 11.64)^‡^
Age								
18-39	1	3808	26.3	0	38391	0	---^§^	---^§^
40-65	14	8346	167.7	11	87400	12.6	13.33 (6.05 - 29.36)^‡^	6.82 (2.96 - 15.69)^‡^
>65	4	1158	345.4	11	14626	75.2	4.59 (1.46 - 14.42)^‡^	3.5 (1.09 - 11.26)*
Medical diseases								
Hypertension								
No	2	2157	92.7	2	93326	2.1	43.27 (6.09 - 307.14)^‡^	46.69 (6.07 - 359.4)^‡^
Yes	17	11155	152.4	20	47091	42.5	3.59 (1.88 - 6.85)^‡^	4.46 (2.29 - 8.7)^‡^
Diabetes mellitus								
No	17	11366	149.6	20	118088	16.9	8.83 (4.63 - 16.86)^‡^	5.28 (2.66 - 10.48)^‡^
Yes	2	1946	102.8	2	22329	9	11.47 (1.62 - 81.46)*	12.31 (1.55 - 98.09)*
Dyslipidemia								
No	12	8668	138.4	17	107454	15.8	8.75 (4.18 - 18.32)^‡^	4.25 (1.92 - 9.39)^‡^
Yes	7	4644	150.7	5	32963	15.2	9.94 (3.15 - 31.31)^‡^	8.7 (2.69 - 28.2)^‡^
Gout								
No	13	9412	138.1	16	122461	13.1	10.57 (5.09 - 21.98)^‡^	8.1 (3.64 - 18.03)^‡^
Yes	6	3900	153.8	6	17956	33.4	4.6 (1.48 - 14.27)^‡^	2.99 (0.96 - 9.38)
Atrial fibrillation								
No	19	12952	146.7	21	138230	15.2	9.66 (5.19 - 17.96)^‡^	5.86 (3.04 - 11.33)^‡^
Yes	0	360	0	1	2187	45.7	---^§^	---^§^
Ischemic heart disease								
No	12	9587	125.2	12	116888	10.3	12.19 (5.48 - 27.14)^‡^	7.61 (3.13 - 18.49)^‡^
Yes	7	3725	187.9	10	23529	42.5	4.42 (1.68 - 11.62)^†^	3.5 (1.3 - 9.43)*
Heart failure								
No	15	11828	126.8	16	134469	11.9	10.66 (5.27 - 21.56)^‡^	6.3 (2.99 - 13.3)^‡^
Yes	4	1484	269.5	6	5948	100.9	2.67 (0.75 - 9.47)	2.54 (0.65 - 9.93)
Cerebrovascular accident								
No	14	11247	124.5	17	129509	13.1	9.48 (4.67 - 19.24)^‡^	5.52 (2.6 - 11.73)^‡^
Yes	5	2065	242.1	5	10908	45.8	5.28 (1.53 - 18.25)^†^	5.74 (1.48 - 22.36)*
Hemorrhagic stroke								
No	17	12580	135.1	20	138646	14.4	9.37 (4.91 - 17.88)^‡^	5.67 (2.85 - 11.27)^‡^
Yes	2	732	273.2	2	1771	112.9	2.42 (0.34 - 17.18)	20.42 (0.63 - 664.32)
Peripheral vascular disease								
No	18	12310	146.2	20	135673	14.7	9.92 (5.25 - 18.75)^‡^	5.94 (3.02 - 11.68)^‡^
Yes	1	1002	99.8	2	4744	42.2	2.37 (0.21 - 26.11)	3.38 (0.21 - 54.62)
Chronic kidney disease								
No	5	6207	80.6	19	137040	13.9	5.81 (2.17 - 15.56)^‡^	5.62 (2 - 15.78)^†^
Yes	14	7105	197	3	3377	88.8	2.22 (0.64 - 7.72)	1.71 (0.47 - 6.19)
Malignancy of kidney or bladder								
No	18	12862	139.9	22	139769	15.7	8.89 (4.77 - 16.58)^‡^	5.38 (2.78 - 10.44)^‡^
Yes	1	450	222.2	0	648	0	---^§^	---^§^

*p < 0.05, ^†^*p* < 0.01, ^‡^*p* < 0.001

^§^Insufficient case number for statistical analysis

Rate denotes incidence rate (per 100,000 person-years).

Adjusted HR (hazard ratio) indicates multivariate analysis with adjusting for age, gender, and comorbidities.

Abbreviations: AAD, aortic aneurysm/dissection; ADPKD, autosomal dominant polycystic kidney disease; PY, person-year; IRR, incidence rate ratio; CI, confidence interval.

It was noteworthy that among hypertensive patients, ADPKD patients had the 4.46-fold higher risk for future development of AAD compared with non-ADPKD patients (95% CI 2.29 to 8.7, *p* < 0.0001). Particularly, as to those normotensive population, ADPKD patients possessed up to 46.69-fold greater risk for AAD than non-ADPKD counterparts (95% CI 6.07 to 359.4, *p* = 0.0002).

### Cox regression analysis for identification of the independent risk factors of AAD (Table 3)

By using the multivariate analysis of Cox regression model with adjustment for age, gender and each of comorbidities, those of male gender, age ≥40 years old, lower urbanization level and hypertension were identified as independent risk factors for future development of AAD (all *p*-values < 0.02). In addition, ADPKD was also a powerful predictor for AAD occurrence (adjusted HR 3.91, 95% CI 1.53 to 10). On the other hand, diabetes mellitus was found to be an independent predictor of free from AAD in ADPKD patients (adjusted HR 0.29, 95% CI 0.1 to 0.84).

**Table 3:** Prediction of occurrence of AAD with Cox proportional hazard regression model

	Univariate			Multivariate		
	HR	95% CI	*p*-value	HR	95% CI	*p*-value
Gender						
Female	1.00			1.00		
Male	2.68	1.37 - 5.25	0.0041	2.75	1.34 - 5.64	0.0058
Age						
18-39	1.00			1.00		
40-65	10.89	1.48 - 80.38	0.0192	8.65	1.13 - 66.01	0.0375
>65	39.67	5.24 - 300.29	0.0004	20.25	2.49 - 164.87	0.0049
Urbanization level						
1 (highest)	1.00			1.00		
2	0.91	0.43 - 1.94	0.814	0.94	0.44 - 2.02	0.8775
3	2.61	1.21 - 5.63	0.0145	2.33	1.04 - 5.22	0.0403
4 (lowest)	0.55	0.07 - 4.23	0.5687	0.41	0.05 - 3.28	0.4033
Income (NTD per month)						
0	1.00			1.00		
1-15,840	0.63	0.23 - 1.75	0.3781	0.62	0.22 - 1.73	0.3625
15,841-25,000	0.57	0.26 - 1.25	0.1587	0.49	0.21 - 1.14	0.0959
>25,000	0.48	0.2 - 1.19	0.1127	0.5	0.18 - 1.38	0.1807
ADPKD						
No	1.00			1.00		
Yes	9.18	4.97 - 16.96	<.0001	3.91	1.53 - 10	0.0043
Medical diseases						
Hypertension	15.01	5.35 - 42.11	<.0001	6.42	2.09 - 19.71	0.0012
Diabetes mellitus	0.57	0.2 - 1.6	0.2875	0.29	0.1 - 0.84	0.0219
Dyslipidemia	1.27	0.65 - 2.49	0.4892	0.83	0.41 - 1.67	0.5918
Gout	2.49	1.27 - 4.87	0.008	0.77	0.37 - 1.6	0.4894
Atrial fibrillation	1.46	0.2 - 10.63	0.7082	0.32	0.04 - 2.41	0.2665
Ischemic heart disease	3.25	1.75 - 6.06	0.0002	1.1	0.54 - 2.22	0.7982
Heart failure	6.29	3.09 - 12.84	<.0001	2.1	0.94 - 4.72	0.0711
Cerebrovascular accident	3.48	1.7 - 7.09	0.0006	0.87	0.35 - 2.17	0.7713
Hemorrhagic stroke	6.49	2.31 - 18.2	0.0004	1.98	0.54 - 7.36	0.3057
Peripheral vascular disease	2.02	0.62 - 6.54	0.2417	0.82	0.25 - 2.71	0.7451
Chronic kidney disease	9.63	5.17 - 17.93	<.0001	1.57	0.61 - 4.03	0.3509
Malignancy of kidney or bladder	3.5	0.48 - 25.48	0.2157	0.77	0.1 - 5.78	0.7981

Abbreviations: AAD, aortic aneurysm/dissection; HR, hazard ratio; CI, confidence interval; NTD, New Taiwan dollars; ADPKD, autosomal dominant polycystic kidney disease.

### Impact of ADPKD and hypertension on the risk of AAD occurrence (Table 4)

We noted that up to 81% of ADPKD patients had co-existing hypertension. In order to further understand the impact of ADPKD and hypertension on the occurrence of AAD, multivariate Cox regression analysis for the risk of ADPKD-associated AAD with interaction of hypertension was performed. After adjusting for age, gender and comorbidities, hypertension and ADPKD were predictive of AAD with adjusted HRs of 13.05 and 30.98 (*p* = 0.0008 and 0.0003), respectively. More importantly, those ADPKD patients with concomitant diagnosis of hypertension had up to 39.85-fold risk for future development of AAD (*p* < 0.0001). These findings suggested that the risk of AAD in ADPKD remarkably increased with the presence of hypertension.

**Table 4:** Cox regression analysis for the risk of ADPKD-associated AAD with interaction of hypertension

	Univariate			Multivariate*		
AAD	HR	95% CI	*p*-value	HR	95% CI	*p*-value
Neither ADPKD nor hypertension	1.00			1.00		
ADPKD only	43.85	6.17 - 311.48	0.0002	30.98	5.39 - 311.40	0.0003
Hypertension only	19.53	4.56 - 83.57	<.0001	13.05	2.92 - 58.30	0.0008
ADPKD and hypertension	71.02	16.41 - 307.40	<.0001	39.85	7.64 - 207.73	<.0001

Abbreviations: ADPKD, autosomal dominant polycystic kidney disease; AAD, aortic aneurysm/dissection; HR, hazard ratio; CI, confidence interval.

*Multivariate analysis was done with adjusting for age, gender, and comorbidities.

### AAD in relation to time since diagnosis of ADPKD as compared with general population (Figure 1)

**Figure 1:**
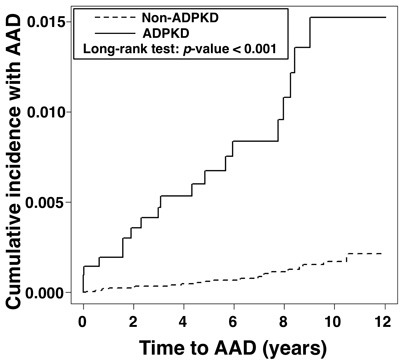
Cumulative incidence of aortic aneurysm/dissection in the ADPKD group *versus* the non-ADPKD group Abbreviation: ADPKD, autosomal-dominant polycystic kidney disease.

The cumulative incidence of AAD between ADPKD and non-ADPKD groups by using Kaplan-Meier analysis is displayed on Figure 1. The Log-Rank test indicated that the cumulative incidence of AAD was significantly higher in patients with ADPKD than in general population (*p* < 0.001). Besides, the mean time period from diagnosis of ADPKD to occurrence of AAD was 4.02±3.16 years. Of note, a time interval of eight years was the critical period for an abrupt increase in the incidence of AAD.

## DISCUSSION

To the best of our knowledge, this is the first nationwide population-based cohort study to establish a strong association between AAD and ADPKD. Our findings demonstrated that the patients with ADPKD had more comorbid diseases than those without ADPKD. Not only was the incidence of AAD significantly higher in the ADPKD than general population, but also the risk of AAD remarkably increased in ADPKD. Importantly, in the present study 58% of AAD patients among ADPKD population were found to have acute aortic dissection. In addition to ADPKD, this study also found that male gender, age of greater than or equal to 40 years, lower urbanization level and hypertension were independent risk factors for future development of AAD. Accordingly, our findings highlight that AAD is a very importantly clinical issue and encourage us routinely to screen and aggressively manage the patients with ADPKD. However, little is known regarding the real-world risk of AAD in ADPKD in the available literature [6, 8, 16], mainly because of insufficient data as compared with other well-recognized complications of ADPKD such as intracranial aneurysms.

Multiple renal and extrarenal cystic formation/growth is the cardinal feature of ADPKD that is mainly resulted from hereditary disorder of PKD1 and PKD2 genes [19, 20]. Studies [21, 22] have further suggested that targeted mutation of either PKD1 or PKD2 could result in disruption of the integrity of polycystin 1 and polycystin 2 in vascular smooth muscle which in turn, cause further dilatation of vascular architecture and ultimately, lead to extensive thoracic and abdominal aneurysm formation. In further, they contribute to the development of hypertension and vascular remodeling, and commonly cause intracranial dolichoectasia and extracranial aneurysms in ADPKD [23, 24]. Intriguingly, despite the documented association between hypertension and vascular aneurysm/remodeling [25, 26], the complex causal relationships among age, hypertension, ADPKD and AAD remain to be clarified. A previous case-control sonographic study [15] that recruited about 300 subjects concluded that patients with ADPKD had only a tendency to develop aortic aneurysms. Accordingly, the ADPKD independently caused high frequency of AAD was questioned by the results of the study [15] for the fact that the higher prevalence of concomitant hypertension and associated connective tissue disorders is also commonly present in AAD. Additionally, another registered data of 426 patients with ADPKD displayed the prevalence of abdominal aortic aneurysm was very low, i.e., about 0.8% [16]. More recently, a review article from Perrone et al. suggested that vascular complications of AAD in ADPKD are uncommon [7]. This issue, therefore, raises a suggestion from a systemic review [27] by Bailey et al. that larger multicenter trials are needed because there has no enough clinical evidence to support a strong association between ADPKD and abdominal aortic aneurysm (AAA). The authors remarked that current available data are insufficient to support for establishing a regular strategy for routine screening of vascular complications in ADPKD [7]. Accordingly, the impact of ADPKD on AAD is still inconclusive. That is the reason why we utilized a nationwide database to do relevant analysis.

In the present study, we found that the prevalence of ADPKD in Asian population is approximately 2.1 in 1,000 Taiwanese individuals (Table 1). Interestingly, the prevalence of ADPKD has been established to be 1 to 2.5 in 1,000 Caucasian general populations [28, 29]. Thus, the prevalence of ADPKD in Asia is very similar to that in the Western countries. We propose that the findings from our study could be transferable to other non-Asian populations.

A principal finding in our study was that ADPKD was a risk factor for future development of AAD shown by multivariate analysis with Cox regression model and stratified analysis. Furthermore, male gender, advancing age and hypertension were also found to be independent risk factors for occurrence of AAD in ADPKD. Moreover, the ADPKD patients had more comorbidities than general population, and those patients with coexistence of ADPKD and hypertension had much high risk for future development of AAD. Another finding was that the frequency of AAD in ADPKD was 0.92% in our Asian cohort study, which was very similar to the reported frequency of 0.8% in a previous smaller ADPKD study from the United States [16]. Taken together, although the frequency of AAD in ADPKD worldwide was around 1%, the risk of AAD substantially increased in ADPKD. ADPKD not only carried an almost 5.5-fold risk for AAD occurrence, but also became much riskier if combined with hypertension. Inevitably, up to eighty percent of ADPKD patients had coexisting hypertension. Therefore, to strictly control blood pressure in ADPKD patients was an important clinical issue for prevention of any vascular complication, especially for AAD. This concept was also supported by current available consensus guidelines [30] that recommend the use of antihypertensive therapies to treat hypertension in ADPKD. The suggested target of blood pressure is less than or equal to 130/80 mmHg.

Our study also demonstrated that the frequency of AAD was eight times higher in the ADPKD than non-ADPKD population (0.92% v.s. 0.11%). A case-review study [13] has shown that type A aortic dissection was remarkably higher than type B aortic dissection (i.e., 62% *vs*. 36%) in patients with ADPKD. In our study, 58% (11/19) of AAD patients had aortic dissection, and rate of thoracic aneurysm was higher than abdominal aneurysm. As a result, our findings and the previous case-review study [13] highlighted that AAD in ADPKD should be viewed as a medical or surgical emergency in the clinical practice. Acute aortic dissection or aneurysmal dissection should always be considered immediately once the ADPKD patients complained of chest or abdominal discomfort. Of particular importance was that the mean time period from ADPKD to AAD was 4.02±3.16 years, suggesting that the first time using image mortalities to follow up these ADPKD patients should be as early as the third year after recognition of ADPKD, followed by regularly image study every year as well as anytime whenever the acute chest/abdominal pain attack.

An interesting finding in the present study was that diabetes mellitus was strong predictive of free from AAD in ADPKD. Intriguingly, the strange relationship between diabetes and AAA had been comprehensively discussed by European Society for Vascular Surgery before [31]. The protective effects of diabetes for AAA has been proposed as the results of slowing aneurysm enlargement and decreasing repairs for aneurysm rupture. In addition, a paradoxical inverse relationship between diabetes and aortic dissection has been found by He et al. in a Chinese population-based case-control study [32]. Another recent finding from Taiwan NHIRD (i.e., a population-based cohort study) has also shown that advanced complicated diabetes mellitus was associated with a reduced risk of thoracic and abdominal aortic aneurysmal rupture [33]. Furthermore, experimental study also has shown that hyperglycemia *per se* rather than its treatment retarded progression of aneurysm [34]. Hence, these clinical and basic studies [31–34] could support the finding of diabetic protection against AAD occurrence in ADPKD in our present study.

### Study limitations and unmet needs

Our study has limitations. First, the NHIRD does not provide detailed personal history and lifestyle information such as smoking, body mass index, and functional capacity. These are potential confounding factors for this study. Second, all the data in the current study have been registered with ICD-9-CM codes, therefore further classification of chronic kidney disease was impracticable. Third, the information of blood pressure and laboratory data are not available in NHIRD. Fourth, we didn't analyze the medication used in study or comparison cohort because technical limitation for data retrieval. Finally, the findings of the present study raise the need of a prospective study to evaluate the potential therapeutic strategies that could prevent or reduce the risk of AAD in ADPKD, therefore, to be as a reference of our clinical practice guideline for these patients in the future.

## CONCLUSIONS

The results of the current study identified that ADPKD patients had substantially higher risk of AAD than comparison cohort.

## MATERIALS AND METHODS

### Data source

The National Health Insurance (NHI) program in Taiwan provides health care to 99% of the 23.74 million and links 97% of the hospitals and clinics in Taiwan [35]. The researchers are able to register and claim data of 1,000,000 individuals who have been systematically selected from all insured enrollees of the National Health Research Institute (NHRI) data bank. The NHI dataset included robust information regarding medical facilities, details of inpatient and outpatient orders, dental services, prescription of drugs, patient care provided by physicians, and the scrambled registration files (e.g., payment, regions, catastrophic illness, and so on) other than laboratory data. Diagnoses are entered in based on the International Classification of Diseases, 9^th^ Revision, Clinical Modification (ICD-9-CM). The Ethics Institutional Review Board of Chang Gung Memorial Hospital approved this study (No.201600124B1).

### Study population

**Figure 2:**
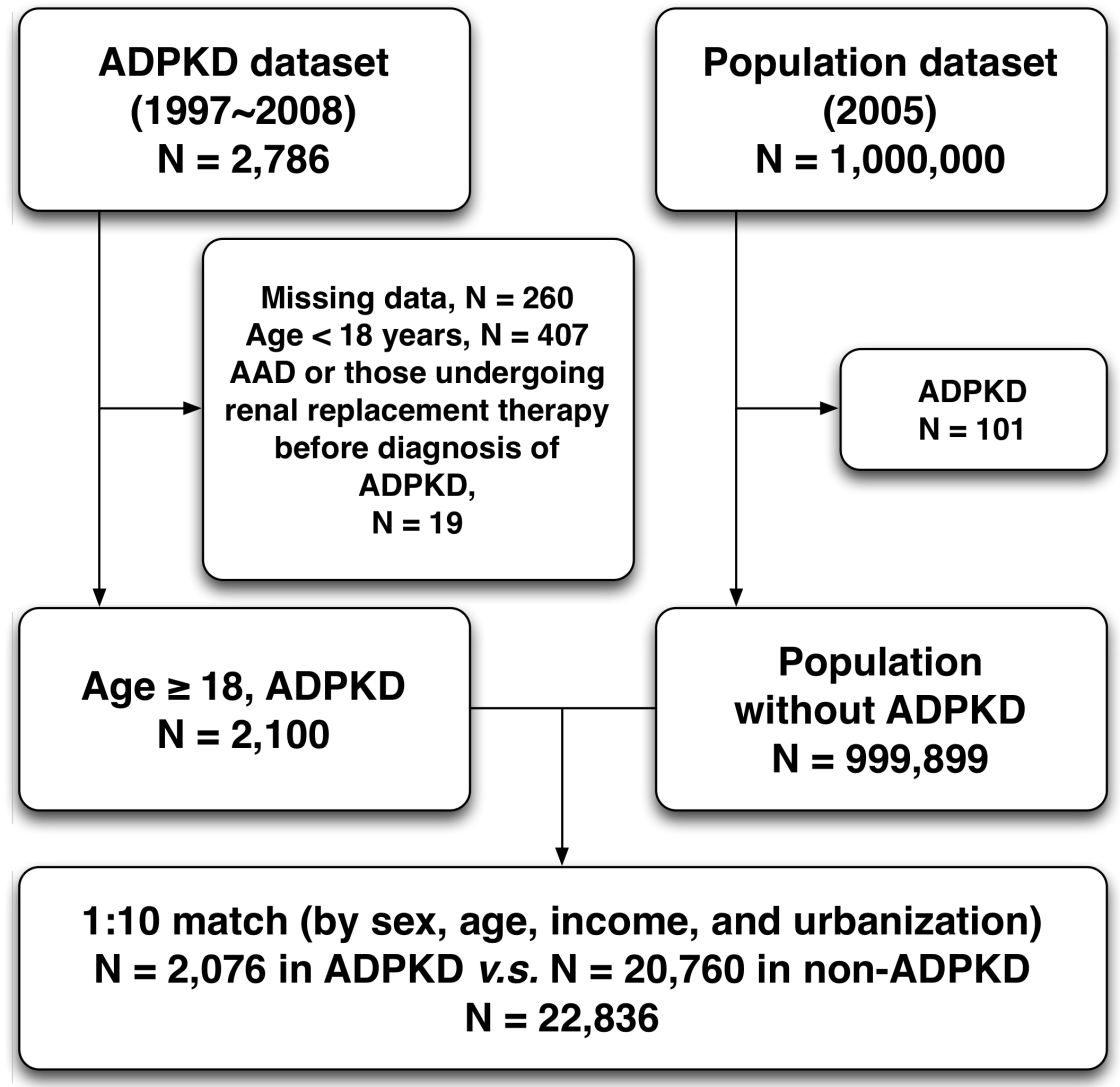
Flowchart of the patient enrollment for the ADPKD group and the matched non-ADPKD group ADPKD = autosomal-dominant polycystic kidney disease, AAD = aortic aneurysm/dissection.

This was a retrospective nationwide population-based cohort study. We selected patients with ADPKD (ICD-9-CM codes: 753.1) from 1,000,000 individuals in Taiwan NHIRD since January 1997 to December 2008. After excluding patients with follow-up duration of less than one year, missing data on baseline characteristics, age less than 18 years old, those receiving renal replacement therapy, and initially concomitant diagnoses of aortic aneurysm/dissection (ICD-9-CM codes 441), a total of 2,076 ADPKD patients were identified. Therefore, the prevalence of ADPKD in Taiwanese population was around 2.1/1,000. The comparison cohort was selected randomly by age-, gender-, income-, and urbanization-matched individuals without history of ADPKD. The ratio of non-ADPKD to ADPKD was 10:1, and therefore 20,760 non-ADPKD patients were allocated into control group (Figure 2). Urbanization of the cities/counties was categorized into four levels (from level 1 indicating the most urbanized to level 4 indicating the least urbanized). The insurance taxable income level per month (expressed by New Taiwan dollars, NTD) was also stratified into four classifications according to monthly salary of individual insured enrollee.

### Outcomes

The diagnoses of ADPKD, aortic aneurysm and aortic dissection were confirmed by consecutive and at least three records of outpatient visits within one year or one diagnosis on admission during study period. We also verified the accuracy of diagnosis of ADPKD by checking the registration of catastrophic illness. The date of the initial diagnosis was defined as the index date. Pre-existing comorbidities for each participant were estimated with hypertension (ICD-9-CM codes 401-405), diabetes (250), dyslipidemia (272), gout (274), atrial fibrillation (427.31), ischemic heart disease (410-414), heart failure (428), cerebral vascular disease (431-436), hemorrhagic stroke (431, 432), peripheral vascular disease (440, 443.9, 444.0, 444.2, 444.8, 444.9, 447.8, 447.9, 445.0, 445.02), chronic kidney disease (585), and malignancy of kidney and bladder (188-189).

The main purpose of this study was to evaluate whether risk of AAD increases in the patients with ADPKD. The frequency and incidence of aortic dissection, aneurysm or aortic aneurysmal dissection (i.e., AAD) were also compared between ADPKD and non-ADPKD groups. In addition, information with respect to the lesion location (thoracic aorta, abdominal aorta, or undefined location) and lesion type (aortic aneurysm, aortic dissection, or coexistence of both conditions) was also analyzed according to ICD-9-CM codes (codes 441.00-441.03, 441.0-441.7 and 441.9). Furthermore, the association between ADPKD and AAD was also analyzed for further identifying real-world risk of AAD occurrence in the ADPKD population.

### Statistical analysis

We compared the distribution of demographic factors and the rate of comorbidities between the study cohort (i.e., ADPKD) and matched control cohort (i.e., non-ADPKD) with the independent t test and Chi-square test. The incidence rate and 95% confidence intervals (95% CI) of AAD were calculated for the entire follow-up period. We utilized the Kaplan-Meier method to estimate cumulative incidences and performed the Log-Rank test to examine differences between disease group and non-disease group in the cohort study. Furthermore, Cox proportional hazard regression models were used to compute the hazard ratios (HRs) and accompanying 95% CIs after adjusting for age, gender, urbanization, income and comorbidities. We also examined the outcome (i.e., occurrence of AAD) stratified by groups according to gender, age, and each of comorbidities. The sensitivity analyses were applied to evaluate the difference and consistency between ADPKD and the risk of AAD. Besides, impact of ADPKD and hypertension on the occurrence of AAD was also analyzed. Two-tailed p-value < 0.05 was considered statistically significant. All the analyses were conducted using SAS statistical software (Version 9.4; SAS Institute, Cary, NC, USA).

## SUPPLEMENTARY MATERIALS TABLE



## Abbreviations

AAD: aortic aneurysm and dissection
ADPKD: autosomal-dominant polycystic kidney disease
CV: cardiovascular
NHIRD: National Health Insurance Research Database
NHRI: National Health Research Institute
HRs: hazard ratios
AAA: abdominal aortic aneurysm
