# Supplementary material for: Risk of aortic aneurysm and dissection in patients with autosomal-dominant polycystic kidney disease: a nationwide population-based cohort study
Source: Oncotarget. 2017 Mar 17;8(34):57594–604. doi: 10.18632/oncotarget.16338 (PMC5593670; doi:10.18632/oncotarget.16338)
Supplement: Supplementary file 1 [file oncotarget-08-57594-s001.pdf]

Risk of aortic aneurysm and dissection in patients with autosomal-dominant polycystic kidney disease: a nationwide population-based cohort study

Supplementary Material

Supplemental Table 1: Lesion type and location of AAD in the patients with and without ADPKD

A) AAD in the ADPKD group (N = 19)

Type	Thoracic	Abdominal	Thoraco-abdominal	Undefined
Aneurysm (N = 8)	4	2	1	1
Dissection (N = 11)	1	1	1	8

B) AAD in the Non-ADPKD group (N = 22)

Type	Thoracic	Abdominal	Thoraco-abdominal	Undefined
Aneurysm (N = 14)	2	10	1	1
Dissection (N = 8)	3	0	0	5
